# Twenty-year sociodemographic trends in lung cancer in non-smokers: A UK-based cohort study of 3.7 million people

**DOI:** 10.1016/j.canep.2020.101771

**Published:** 2020-08

**Authors:** Greta Rait, Laura Horsfall

**Affiliations:** Research Department of Primary Care and Population Health, University College London, Royal Free Hospital Campus, London, NW3 2PF, United Kingdom

**Keywords:** CI, Confidence Interval, LCINS, lung cancer in non-smokers, PYs, person years, THIN, The Health Improvement Network, UK, United Kingdom, Lung cancer, Epidemiology, Cohort study, Never smokers, Electronic healthcare records

## Abstract

**Introduction:**

: There are reports that lung cancer in non-smokers (LCINS) is increasing in the United Kingdom (UK) and other high-income countries but evidence from large-scale cohort studies to support this claim is limited.

**Material and methods:**

: Using The Health Improvement Network (THIN) IQVIA™ Medical Research Data, we identified a cohort of 3,679,831 people from the UK self-reporting to their primary care physician as never or non-smokers. We estimated age-adjusted incidence rates for recorded lung cancer before (1998−2007) and after (2008−2018) the introduction of smoke-free legislation using multivariable Poisson regression. We also explored the impact of geographic location, social deprivation and urbanicity.

**Results:**

: The analysis included 3,212 lung cancer events and 28 million person-years (PYs). Between 1998 and 2007, the age-adjusted rates in men declined by 9% per year (95 %CI: 7–11%) from an estimated 5.6 to 1.5 per 10,000 PYs and by 3% per year (95 %CI: 1–5%) between 2008 and 2018. These trends for men were similar across sociodemographic strata. Between 1998 and 2007, age-adjusted rates were stable for women at 1.5 per 10,000 PYs. However, there was evidence that time trends for women differed depending on levels of social deprivation with rates increasing by 5% per year (95 %CI: 2–9%) from an estimated 1.3–2.1 per 10,000 PYs for women living in the least socially deprived areas. Sex-specific time trends from 2008 to 2016 were broadly similar in a separate cohort of self-reported never smokers from UK Biobank with cancer events linked to national registries.

**Conclusion:**

In summary, the incidence of LCINS has reduced or remained stable for most of the UK with the possible exception of women living in the least socially deprived areas.

## Highlights

• This study of 3.7 million people was prompted by concerns that lung cancer in non-smokers (LCINS) is increasing in the UK.
• Using a cohort approach, we could account for the increase in never smokers over time as well as the aging population.
• Our results suggest that the incidence of LCINS over the past twenty years has reduced dramatically in men and remained fairly stable in women.

## 1 Introduction

Lung cancer is responsible for the largest number of cancer deaths worldwide (1.8 million deaths, 18.4 % of the total) [1]. Cigarette smoking is the main risk factor for lung cancer but as fewer people smoke more cases will be diagnosed in people who have never smoked or have smoked fewer than 100 cigarettes in their lifetime. In the UK for example, smoking prevalence has decreased in men from 65 % in 1948 to 19 % in 2017 and in women from 41 % in 1948 to 15 % in 2017 [2]. A recent study in one large UK hospital during 2014, found that self-reported never smokers accounted for as many as 27 % of lung cancer cases [3]. Known risk factors for lung cancer in never smokers are exposure to second-hand smoke, environmental pollution, occupational carcinogens, radon, infections and genetic factors [4]. Exposure to occupational and environmental carcinogens has decreased in the UK since the 1970s with far fewer people working in primary sectors jobs (e.g. mining), tighter occupational regulation and improved ambient air quality. More recently, the UK countries introduced legislation to ban smoking in workplaces and enclosed public places starting in Scotland. By July 2007, all countries had implemented the smoke free legislation. The health effects were immediate with measures of cotinine levels decreasing by around 27 % on average [5], hospital admissions for asthma falling by 5.0 % and heart attacks falling by 2.4 % [6,7]. Further legislation followed including an increase in the minimum age of sale to 18 years in 2015 and the introduction of “plain” tobacco packaging in 2016.

Despite these long- and short-term reductions in known risk factors, there are recent reports that LCINS is increasing in the UK [3,8]. An increase in LCINS incidence would be worrying from a public health perspective particularly given growing concerns in the UK regarding the dramatic increase in harmful emissions from domestic wood combustion over the past 15 years [9]. However, it is unclear from the available evidence whether the reported increase in LCINS simply reflects a combination of an increase in never smokers and the ageing population. Furthermore, there are limited data available for more recent time periods.

Using electronic health care records from the UK primary health care setting, we identified a cohort of 3.7 million self-reported non-smokers and explored trends in lung cancer incidence over the past 20-years. Using a cohort approach, we could account for the increase in never smokers over time as well as the ageing population. Occupational and environmental risk factors for LCINS could vary by sex at birth, levels of social deprivation, urban versus rural living, and UK country/geographic region and we therefore explored these relationships with total rates and time trends. We additionally compared time trends with those of a smaller but highly characterised cohort from UK Biobank with lifetime smoking behaviour recorded and cancer outcomes linked with national cancer registry data.

## 2 Material and methods

### 2.1 Data sources

The data source is The Health Improvement Network (THIN) supplied by IQVIA™ Medical Research Data [10]. This dataset contains de-identified routinely collected electronic patient health record data supplied from UK General Practitioner (GP) computer systems using the VISION software. THIN data covers around 6% of the UK population and includes information on symptoms, prescriptions, immunisations, lab test results, health behaviours and other postcode (zip code) linked sociodemographic variables. These data are broadly representative of the UK population and diagnoses of a broad range of conditions are comparable to other reliable sources [11]. The data set we used had information recorded up to January 2019.

Previous studies of primary care data have shown that lung cancer is underreported relative to the national cancer registries [12]. Further, we only have a patients’ smoking history for the duration they are registered with the general practice. Therefore we compared trends in LCINS from THIN with UK Biobank data from 2008 to 2016 (https://www.ukbiobank.ac.uk/). This cohort of half-a-million UK participants includes information on lifetime smoking behaviour and contains cancer outcomes linked to national registries. Further details are included in the appendices.

The use of IQVIA™ Medical Research Data for the purpose of medical research and for supplying the data to external researchers for scientifically approved studies under Data Sharing Agreements has been approved by the NHS Health Research Authority (NHS Research Ethics Committee ref 18/LO/0441). The protocols for the present study were approved by the IQVIA scientific review committee in August 2019 (ID:19THIN048) and by UK Biobank in December 2019 as part of a scope expansion to an existing project (ID:5167).

### 2.2 Study design

We designed a cohort study where patients entered the study at the latest date of GP practice registration plus six months [13], 18th birthyear, and after the practice met electronic recording quality criteria [14,15]. Patients exited the cohort at the earliest date of transferring to a different GP practice, death, lung cancer diagnosis, 100th birthyear, end of the study period (December 31st, 2018), and the practice stopped contributing data to THIN.

### 2.3 Inclusion criteria

We included patients between the ages of 18 and 100 who contributed at least one year of acceptable quality follow-up data [14,15]. Time periods before January 1 st, 1998 and after study end date of 31st of December 2018 were excluded. Primary care physicians in the UK record symptoms, diagnosis and health behaviour such as smoking using a hierarchical coding system called Read codes [16]. We used a published method to identify codes for smoking status from the medical records [17] (Table A.1). The majority of smoking data is recorded by GPs using a structured data template with smokers coded as “Never”, “Former” or “Current”. For each patient, we applied an algorithm to the Read codes and structured data template to exclude current or ex-smokers and those with no smoking data. Patients with prescriptions for nicotine replacement therapy in their drug prescription records were also excluded.

### 2.4 Outcome

We used a published method to identify Read codes for lung cancers and applied these to the medical and death records for each patient [17] (Table A.2). The diagnosis was considered an incident case if it was recorded at least six months after the patient registered with the GP and was therefore less likely to represent the health professional coding a medical history [13].

### 2.5 Covariates

The main covariate of interest was calendar year. We also explored whether trends over time differed by sex at birth, socioeconomic status, rural-urban classification and geographic region of the UK. Social deprivation is defined using a composite measure of unemployment, non-car ownership, non-home ownership and overcrowding (Townsend score) [18]. The data provider categorises the Townsend score into quintile categories of deprivation before the data are released. Geographic region relates to the boundaries of the former strategic health authorities based in England. Urbanicity is provided using the UK Government classification system [19].

### 2.6 Statistical analyses

Incidence rates per 10,000 person years were calculated with 95 % confidence intervals assuming a Poisson distribution. We used attained age as the timescale and data were spilt by one-year intervals for age and calendar year. Age and calendar year were parameterised as continuous variables and we used multivariable Poisson regression analyses to estimate incidence rate ratios and the marginal effects (average predicted incidence rates) adjusted for age and other covariates where appropriate. In contrast to the rate ratio scale that has a baseline comparator, the marginal effect is an estimation of how much the incidence rate is expected to change for a unit change in an explanatory variable and is useful for visualising interaction effects that are hard to interpret directly from the model coefficients. We calculated the marginal effects for fixed values of calendar year holding all other variables in the model at their observed values and using the delta method for estimating standard errors. We fitted a piecewise linear function for calendar year with a knot placed at 2008 to estimate the average incidence change per year before and after the introduction of the UK smoke-free legislation. We used the Akaike’s Information Criterion (AIC) to check whether further knots or cubic spline transformations improved model fit without adding unnecessary complexity. Wald tests were used to calculate p-values for categorical variables and multiplicative interaction terms. Observations are clustered within GP practices and therefore we included the practice identifier to estimate robust standard errors. We checked for overdispersion by running negative binomial models and comparing outputs. All statistical analyses were conducted using Stata v.16.1 (Stata Corporation, College Station, Texas).

## 3 Results

We identified 8,992,142 people aged between 18 and 100 years contributing at least one year of acceptable quality person-year data. After excluding 4,393,786 (48 %) ever smokers, 604,768 (7%) with no smoking data, we were left with 3,993,588 (44 %) self-reported never/non-smokers. A cohort of 3,679,831 people remained after excluding those who exited the cohort within six months of joining the GP practice or only contributed data prior to 1998. The final analysis included 3,212 lung cancer events and 28 million person years of acceptable quality data (Table 1). We used the full data with interaction terms to estimate the overall sex-specific time trends and ran analyses separately by sex to examine interactions between calendar year and other variables.

**Table 1:** Cohort characteristics and unadjusted incidence rates for lung cancer in non-smokers by sociodemographic factors and stratified by sex at birth.

	Women			Men		
Number	2,088,590			1,591,241		
Median follow-up (IQR)	6 (3–12)			6 (3–12)		
Mean age at start	41.9 (19.7)			37.9 (16.8)		
	Events	PYs (10,000)	Incidence rate (95 %CI)	Events	PYs (10,000)	Incidence rate (95 %CI)
	1879	1600	1.2 (1.1–1.2)	1333	1240	1.1 (1–1.1)
Time period						
1998-	271	230	1.2 (1.1–1.3)	347	160	2.1 (1.9–2.4)
2003-	626	530	1.2 (1.1–1.3)	457	400	1.2 (1.1–1.3)
2008-	467	390	1.2 (1.1–1.3)	247	310	0.8 (0.7 to 0.9)
2013-	515	450	1.2 (1.1–1.3)	282	370	0.8 (0.7 to 0.9)
Age						
<40	16	550	0 (0 to 0.1)	21	520	0 (0 to 0.1)
40	71	300	0.2 (0.2 to 0.3)	38	270	0.1 (0.1 to 0.2)
50	163	260	0.6 (0.5 to 0.7)	109	210	0.5 (0.4 to 0.6)
60	357	210	1.7 (1.6–1.9)	249	130	1.9 (1.7–2.2)
70	565	160	3.6 (3.3–3.9)	483	76	6.4 (5.8–7.0)
80	578	100	5.6 (5.2–6.1)	365	33	10.9 (9.8–12.1)
90+	129	25	5.1 (4.2–6.0)	68	5	13.6 (10.6–17.2)
Townsend score						
1 (least deprived)	452	400	1.1 (1.0–1.2)	351	310	1.1 (1.0–1.2)
2	409	330	1.2 (1.0–1.3)	284	250	1.3 (1.1–1.4)
3	314	280	1.1 (0.9–1.2)	224	210	1.1 (1.0–1.3)
4	253	210	1.2 (1.0–1.4)	190	160	1.2 (1.0–1.3)
5 (most deprived)	169	130	1.3 (1.0–1.5)	123	98	1.3 (1.1–1.6)
Missing	282	250	0.8 (0.7 to 0.9)	161	200	1.1 (1.0–1.2)
Urban-rural						
1 = Urban >10k – Sparse,	5	2.3	2.2 (0.7–5.1)	2	1.5	0.8 (0.7 to 0.9)
2 = Town & Fringe – Sparse,	10	6.0	1.7 (0.8–3.1)	8	4.5	1.3 (0.2–4.7)
3 = Village, Hamlet & Isolated dwellings – Sparse,	8	7.8	1.0 (0.4–2.0)	4	6.2	1.8 (0.8–3.5)
4 = Urban >10k - Less sparse,	1045	870	1.2 (1.1–1.3)	770	660	0.7 (0.2–1.7)
5 = Town & Fringe – Less sparse	181	130	1.4 (1.2–1.6)	151	99	1.2 (1.1–1.3)
6 = Village, Hamlet & Isolated dwelling – Less sparse.	92	76	1.2 (1.0–1.5)	65	58	1.5 (1.3–1.8)
Missing	538	510	1.1 (1.0–1.2)	333	410	0.8 (0.7 to 0.9)
Country						
England	1339	1100	1.2 (1.1–1.3)	981	840	1.2 (1.1–1.2)
Northern Ireland	87	76	1.1 (0.9–1.4)	49	60	0.8 (0.6–1.1)
Scotland	229	230	1.0 (0.9–1.1)	151	190	0.8 (0.7 to 0.9)
Wales	224	180	1.2 (1.1–1.4)	152	150	1.0 (0.9–1.2)
Regions of England						
East Midlands	48	39	1.2 (0.9–1.6)	48	29	1.7 (1.2–2.2)
East of England	96	92	1.0 (0.8–1.3)	76	69	1.1 (0.9–1.4)
London	191	190	1.0 (0.9–1.1)	132	150	0.9 (0.8–1.1)
North East	38	27	1.4 (1.0–1.9)	36	21	1.7 (1.2–2.3)
North West	214	140	1.5 (1.3–1.7)	157	110	1.5 (1.2–1.7)
South Central	215	170	1.3 (1.1–1.4)	134	130	1.0 (0.9–1.2)
South East Coast	178	160	1.1 (1.0–1.3)	124	120	1.1 (0.9–1.3)
South West	152	120	1.3 (1.1–1.5)	114	85	1.3 (1.1–1.6)
West Midlands	152	140	1.1 (0.9–1.3)	116	100	1.1 (0.9–1.3)
Yorkshire & Humber	55	36	1.5 (1.2–2.0)	44	27	1.6 (1.2–2.2)

IQR = Interquartile range.

### 3.1 Time trends by sex at birth

**Fig. 1:**
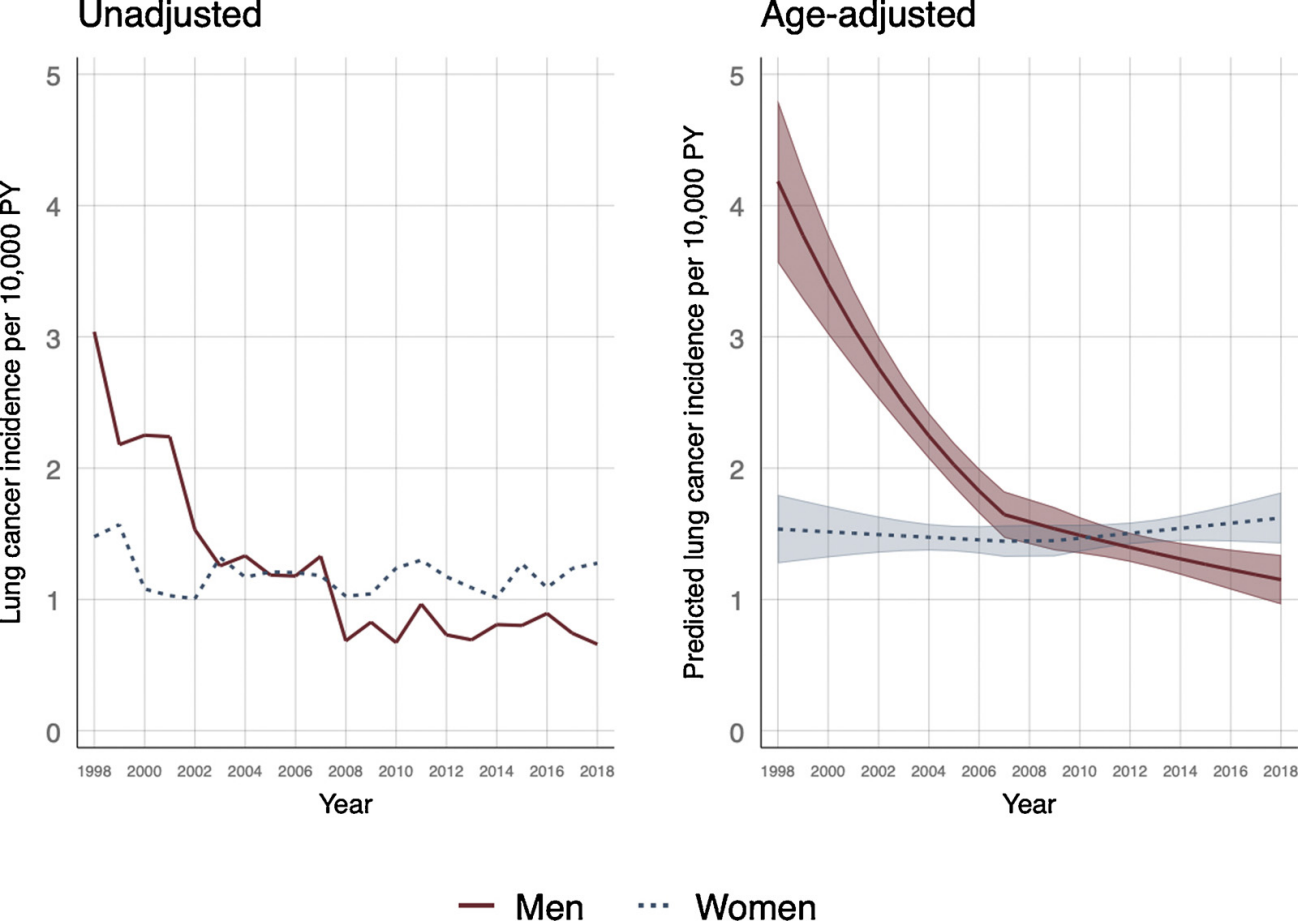
Unadjusted (left) incidence and age-adjusted predicted incidence (right) of lung cancer in non-smokers by calendar year and sex at birth with 95 % confidence intervals.

For women, the age-adjusted incidence rates have been relatively stable over the past 20-years at around 1.5 per 10,000 PYs (Table 2, Fig. 1). Between 1998 and 2008, age-adjusted incidence rates in men decreased by 9% per year on average and by 3% per year thereafter (Table 2, Fig. 1). The predicted incidence rate decreased by around 5.5 per 10,000 PYs to 2.2 per 10,000 PYs in the ten years from 1998 (Fig. 1). By 2018, age-adjusted LCINS rates in men were estimated to be lower than women (Fig. 1).

**Table 2:** Age-adjusted incidence rates and incidence predictions for lung cancer in non-smokers by UK geographic region and sex at birth.

	Age-adjusted IRR (95 %CI)	P-value*	Predicted age adjusted incidence per 10,000 PY	IRR change per year (95 %CI)	IRR change per year (95 %CI)	P-value**
				1998−2007	2008−2018	
Women	1 (ref)		1.50 (1.43–1.57)	0.99 (0.97–1.02)	1.01 (0.99–1.03)	
Men	1.62 (1.50–1.75)	<0.0001	1.83 (1.71–1.95)	0.91 (0.89 to 0.93)	0.97 (0.95 to 0.99)	<0.0001
Women						
England	1 (ref)		1.51 (1.43–1.60)	0.99 (0.97–1.01)	1.02 (1.00–1.05)	
Northern Ireland	0.97 (0.77–1.21)		1.47 (1.15–1.79)	1.03 (0.93–1.13)	1.03 (0.96–1.10)	
Scotland	0.91 (0.80–1.04)		1.38 (1.21–1.55)	1.01 (0.93–1.11)	0.96 (0.92–1.01)	
Wales	1.05 (0.91–1.20)	0.46	1.58 (1.39–1.78)	0.99 (0.93–1.05)	1.03 (0.98–1.08)	0.21
Men						
England	1 (ref)		1.93 (1.79–2.07)	0.91 (0.88 to 0.93)	0.98 (0.95–1.01)	
Northern Ireland	0.73 (0.52–1.03)		1.42 (0.95–1.88)	0.88 (0.78 to 0.98)	0.92 (0.85–1.0)	
Scotland	0.79 (0.65 to 0.95)		1.52 (1.26–1.78)	0.91 (0.85 to 0.97)	0.96 (0.91–1.02)	
Wales	0.92 (0.75–1.13)	0.028	1.78 (1.44–2.13)	0.91 (0.86 to 0.97)	0.98 (0.92–1.04)	0.72

IRR = Incidence rate ratio.

* Wald test for categorical variable.

** Wald test for multiplicative interaction term between variable and calendar year.

**Fig. 2:**
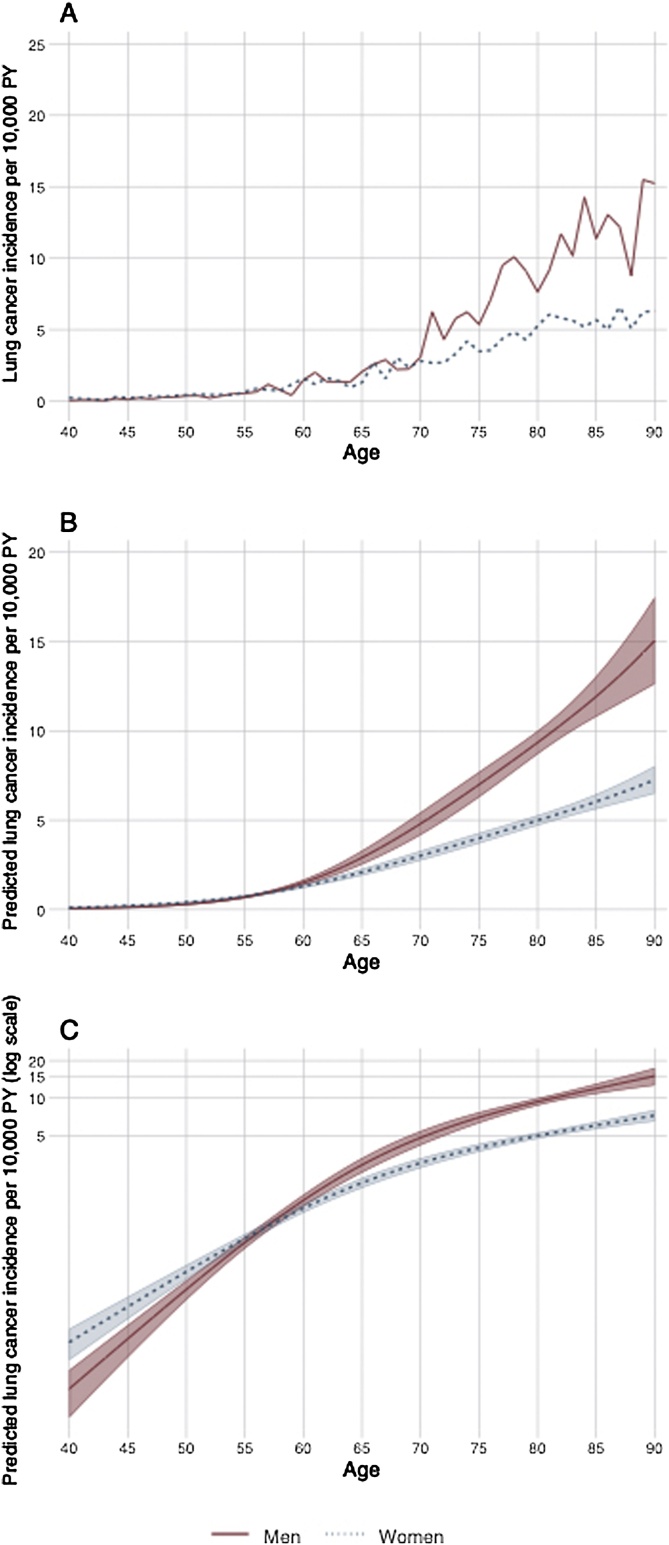
Relationship between age and of lung cancer in non-smokers by sex (three-knot cubic spline transformation) showing unadjusted rates (A), predicted incidence rates adjusted for calendar year (B) and on the log scale to visualise differences at lower incidence rates at younger ages (C).

**Fig. 3:**
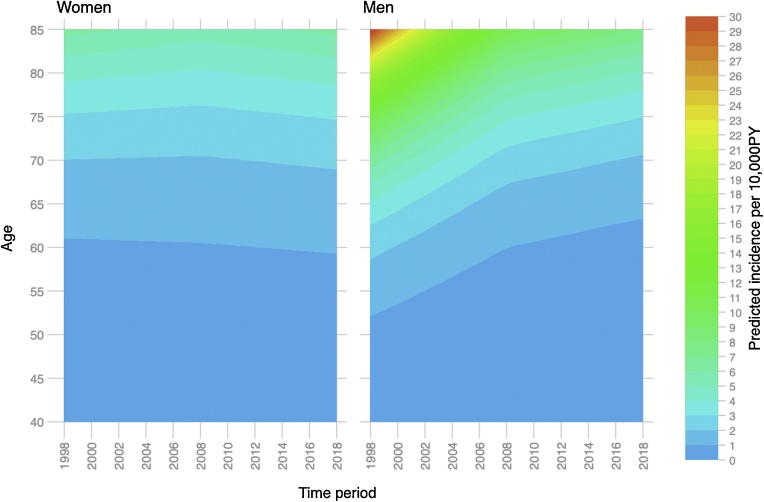
Predicted incidence of lung cancer in non-smoking men and women by age and calendar year (Wald test for interaction terms P = 0.35 for women and P = 0.002 for men).

LCINS incidence rate was higher for women until around age 50 (Table 1, Fig. 2). There was no strong evidence that incidence by age has changed in women over time (Wald test for interaction term P = 0.35) (Fig. 3). Compared with younger men the incidence in older groups have seen larger deceases over time (Wald test for interaction term P = 0.002) (Fig. 3).

### 3.2 Social deprivation

**Fig. 4:**
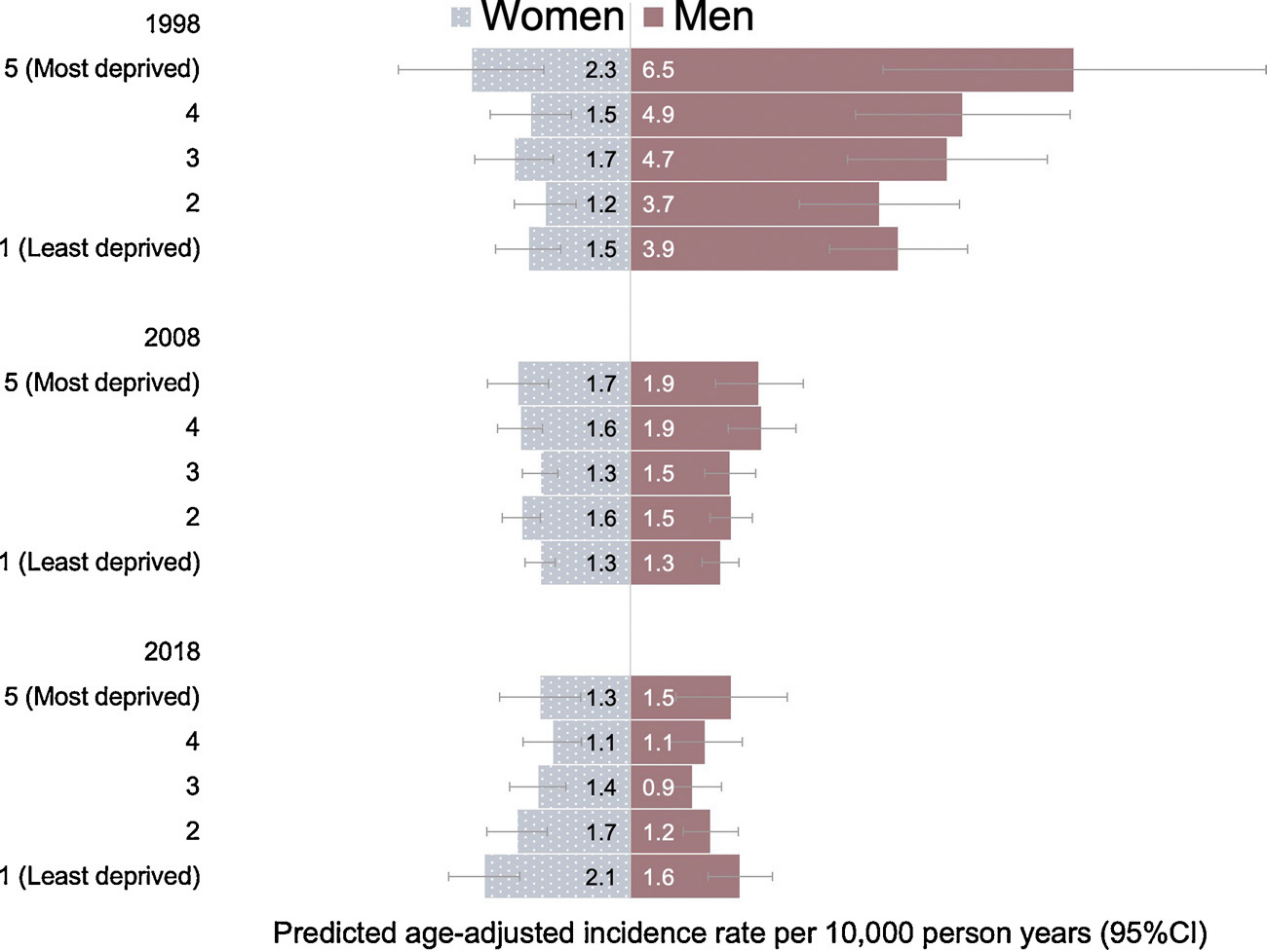
Predicted age-adjusted incidence with 95 % confidence intervals of lung cancer in non-smokers at three time points in the past 20-years by sex at birth and quintile of Townsend measure of social deprivation.

The incidence of LCINS was higher in the most socially deprived areas for both sexes (Table 1). Although, age-adjusted incidence rates were not very different across levels of social deprivation for women, there was some evidence of an interaction with calendar year (Table 3). Between 1998 and 2008, incidence rates were stable for women but from 2008 LCINS increased by around 5% per year (95 %CI: 2–9%) for those living in the least socially deprived areas (Table 3, Fig. 4). Rates declined in both time periods for women in the most socially deprived areas. The predicted incidence rates suggest trends across levels of social deprivation may have reversed since 1998 with rates for women living in the least socially deprived areas estimated to be 60 % higher than the most deprived in 2018 (Fig. 4). After adjusting for age, rates in men were 35 % (95 %CI: 11–68%) higher in the most socially deprived quintile compared with the least deprived quintile (Table 3, Fig. 4). Over time, LCINS in men has decreased at a similar rate across most levels of social deprivation.

**Table 3:** Age-adjusted incidence rates and incidence predictions for lung cancer in non-smokers by social deprivation/urban-rural classification and sex at birth.

Women						
Townsend score	Age-adjusted IRR (95 %CI)	P-value*	Predicted age adjusted incidence per 10,000 PY	IRR change per year 1998−2007 (95%CI)	IRR change per year 2008−2018 (95%CI)	P-value**
1 (least deprived)	1 (ref)		1.52 (1.37–1.67)	0.98 (0.98–1.07)	1.05 (1.02–1.09)	
2	1.01 (0.88–1.16)		1.54 (1.39–1.69)	1.02 (0.98–1.07)	1.00 (0.97–1.04)	
3	0.92 (0.79–1.07)		1.40 (1.25–1.56)	0.97 (0.93–1.02)	1.00 (0.96–1.05)	
4	0.98 (0.83–1.15)		1.48 (1.29–1.67)	1.01 (0.96–1.07)	0.96 (0.92–1.01)	
5 (most deprived)	1.13 (0.94–1.36)	0.31	1.72 (1.46–1.98)	0.97 (0.91–1.03)	0.98 (0.92–1.04)	0.021
Urban-rural						
Urban	1 (ref)		1.53 (1.44–1.63)	0.99 (0.96–1.01)	1.01 (0.99–1.04)	
Village	0.96 (0.82–1.12)		1.47 (1.27–1.68)	0.99 (0.96–1.01)	1.06 (1.00–1.12)	
Rural	0.91 (0.74–1.11)	0.59	1.39 (1.12–1.66)	1.00 (0.92–1.08)	1.07 (0.99–1.15)	0.27
Urban-rural adjusted for Townsend						
Urban	1 (ref)		1.56 (1.46–1.66)	0.99 (0.94–1.03)	1.04 (1.00–1.08)	
Village	0.98 (0.83–1.14)		1.52 (1.30–1.74)	0.99 (0.94–1.03)	1.09 (1.02–1.16)	
Rural	0.92 (0.74–1.13)	0.70	1.43 (1.14–1.72)	0.99 (0.91–1.08)	1.10 (1.01–1.19)	0.45
Men						
Townsend score	Age-adjusted IRR (95 %CI)	P-value	Predicted age adjusted incidence per 10,000 PY	IRR change per year 1998−2007 (95%CI)	IRR change per year 2008−2018 (95%CI)	P-value
1 (least deprived)	1 (ref)		1.78 (1.59–1.97)	0.90 (0.87 to 0.96)	1.02 (0.98–1.07)	
2	0.98 (0.84–1.15)		1.75 (1.53–1.96)	0.91 (0.87 to 0.96)	0.98 (0.93–1.03)	
3	1.01 (0.85–1.20)		1.80 (1.54–2.06)	0.89 (0.85 to 0.94)	0.95 (0.89–1.01)	
4	1.24 (1.04–1.48)		2.22 (1.90–2.53)	0.91 (0.87 to 0.96)	0.94 (0.88–1.01)	
5 (most deprived)	1.42 (1.15–1.76)	0.0019	2.53 (2.04–3.01)	0.88 (0.83 to 0.95)	0.98 (0.91–1.05)	0.32
Urbanicity						
Urban	1 (ref)		2.02 (2.17 to 2.04)	0.90 (0.87 to 0.92)	0.99 (0.96–1.02)	
Village	1.03 (0.87–1.22)		2.09 (2.42 to 1.94)	0.89 (0.84 to 0.94)	1.03 (0.96–1.11)	
Rural	0.71 (0.56 to 0.91)	0.021	1.44 (1.78–1.84)	0.94 (0.86–1.02)	0.96 (0.86–1.07)	0.61
Urban-rural adjusted for Townsend						
Urban	1 (ref)		2.04 (1.89–2.19)	0.90 (0.86 to 0.94)	1.01 (0.96–1.06)	
Village	1.09 (0.92–1.30)		2.23 (1.87–2.58)	0.89 (0.84 to 0.95)	1.06 (0.98–1.14)	
Rural	0.78 (0.61–1.01)	0.07	1.60 (1.21–1.98)	0.94 (0.86–1.03)	0.98 (0.88–1.10)	0.67

IRR = Incidence rate ratio.

* Wald test for categorical variable.

** Wald test for multiplicative interaction term between variable and calendar year.

### 3.3 Urbanicity

Due to low numbers of events, we combined the six original categories into towns, village and rural areas for the analyses. Compared with towns, the age-adjusted incidence rates of LCINS in rural areas were 9% lower for women and 30 % lower for men (Table 3). Time trends were similar across towns, villages and rural areas for men and women (Table 3). Adjusting for differences in social deprivation reduced the strength of the associations but did not alter the overall findings.

### 3.4 Geographic variation

The age-adjusted incidence rates of LCINS for women were broadly similar across UK countries (Table 2). In men, incidence rates in England were around 20–30 % higher than other countries (Table 2). The age-adjusted reductions in LCINS incidence in men up to 2007 were largely driven by England and Wales with more steady declines in Northern Ireland and Scotland over the 20-years (Table 2, Figure A.1).

There was evidence of regional variation in England for overall rates and for time trends (Table A.3, Figure A.2). Overall, the North of England had the highest age-adjusted rates of LCINS for men and women (Table A.3). Time trends were broadly similar across English regions for women with the possible exception of the North East, which saw a 15 % decrease in incidence between 1998 and 2007 (95 %CI: 7–22 %) (Table A.3, Figure A.2). Between 1998 and 2007, the average annual decrease in LCINS in men ranged from 2 to 15% per year with only Yorkshire and Humber showing no reduction (Table A.3, Figure A.2).

### 3.5 UK Biobank time-trends

There were 354 lung cancer events and 186,000 PYs in self-reported never smokers in UK Biobank. The age-adjusted predicted incidence of LCINS was similar to those for THIN from 2008. (Figure A.3, supplementary results). Estimated trends were mostly compatible with an increase per year in women (IRR: 1.04 [95 %CI: 0.97–1.11]) and decrease per year in men (IRR: 0.93 [95 %CI: 0.85–1.01]) since 2008 but these estimates were imprecise due to low numbers of events. Due to low events we did not explore other sociodemographic variables.

## 4 Discussion

### 4.1 Summary

This study was prompted by recent concerns that LCINS is increasing in the UK [8]. Overall, our results suggest that the incidence of LCINS has reduced or remained fairly stable for most of the UK. We found that earlier time periods, high levels of social deprivation, living in an urban environment, and living in the North of England were associated with higher age-adjusted LCINS rates in men. Between 1998 and 2008, the age-adjusted rates in men declined across most sociodemographic and geographic strata. On the other hand, rates were stable for women overall with some evidence of an increase since 2008 that seemed to be driven by women living in the least socially deprived areas. The results for UK Biobank were mostly compatible with an increase since 2008 for women and a decrease for men but these estimates were imprecise due to low events.

Although sex-specific time trends in LCINS incidence could be explained by sex-specific changes in diagnosis or smoking misclassification, we believe changes in environmental risk factors have played an explanatory role. Fon instance in 1966, around 45 % of the UKs predominantly male workforce worked in primary and secondary sector jobs (agriculture, mining and manufacturing) compared with 16 % by 2016 [20]. These sectors are associated with higher levels of exposure to major lung carcinogens including asbestos, silica, certain pesticides and diesel fumes relative to tertiary sector jobs [21]. The attributable fraction for lung cancer due to occupational carcinogens is high in men ranging from 10 to 30% compared to 1–5 % for women [21]. Changes in ambient air quality may also contribute to the downward trend in LCINS in men. Between 1998 and 2018 in the UK, ambient nitrogen dioxide decreased by around 40 to 20 μg/m³ in urban background and 15 to 8 μg/m³ in rural areas [22]. With higher historic rates of employment and full-time working, improvements in outdoor air-quality could also have led to a stronger risk reduction in men relative to women. Furthermore, reductions in second-hand smoke from smoke free legislation at work and when socialising could have had a stronger impact on men than women.

However, it remains surprising that the reductions in the major risk factors for LCINS have had no impact on women. One possibility is that any gains for women have been offset by other harmful environmental exposures. Interestingly, we found that LCINS incidence was increasing for women living in the least deprived areas. There has been growing concern in the UK over emissions of harmful particulates (PM2.5) from domestic wood burning, which have more than doubled between 2003 and 2018 (from 20 to 41 thousand tonnes) and by 6.8 % between 2017 and 2018 alone [9]. Woodburning stoves are high-cost items that are unsuitable for high-density housing and are therefore more prevalent in the least socially deprived areas. Women in the UK spend more time at home on average compared to men and have greater exposure to domestic combustion products. Therefore, increased woodburning could contribute to the upward time-trends in LCINS rates in the least socially deprived women. There is robust evidence that indoor air pollution from coal burning causes lung cancer and is classified by the International Agency for Research on Cancer (IARC) as a Group 1 carcinogen in 2010. However, there are fewer conclusive studies for woodburning, which led to an IARC Group 2A carcinogen assignment. Over the past 40 years, the proportion of women in paid employment in the UK has increased from 57 % in 1975 to 78 % in 2017 [23]. Entering the workforce could be associated with higher exposure to outdoor air pollution (e.g. diesel fumes) and occupational carcinogens and could also explain the upward trend in women in the least socially deprived areas. Our observed time-trends for social deprivation could also reflect chance findings, differences in smoking misclassification, symptom recognition, diagnosis or temporal changes in unmeasured variables such as ethnicity across levels of social deprivation.

### 4.2 Comparison with other studies

To the best of our knowledge, this is the largest analysis of LCINS in recent years for a single nation. We are aware of one other large-scale UK-based cohort study that analysed LCINS in subset of 634,039 women from UK Million Women Study [24]. Unlike our study, lung cancer cases were defined using hospital registries. The LCINS rates for women aged 60–69 according to their supplementary data were similar to ours at 1.7 per 10,000 person years. Although time trends were not examined, the study found that out of 31 potential risk factors, just asthma, height and ethnicity were associated with LCINS. The incidence rate in the most deprived tertile was 12 % higher than the least deprived which was similar to our findings of 13 % for quintiles of deprivation. Due to LCINS being relatively rare, most other large-scale studies have pooled data across countries for earlier time periods and are difficult to compare directly to our findings. One of the largest analyses of LCINS was a pooled analysis of 13 cohorts with participants from the US and Europe (376,600 women, 253,600 men and 4795 incident cases) [25]. However, this study was focussed on age trends and data for temporal analyses were limited. The overall age-adjusted rates for the pooled analysis were similar for men and women although rates were higher in women aged 40–59 years. For the subset of data linked to time period up to 2004, there was no indication that the incidence or death from LCINS has changed for people aged between 40–70 years of age in the US since the 1930s. A separate analysis of data from six large cohorts with data up to 2002 estimated age-adjusted LCINS incidence ranged from 1.4 to 2.1 per 10,000 person-years for women and 0.5–1.4 per 10,000 person-years for men [26]. This study could not assess trends over time.

Large-scale global analyses of overall lung cancer incidence suggest that lung cancer rates in women are increasing and seem to be overtaking those of men in many high-income countries, which some have argued is inconsistent with smoking trends and suggests a potential role for other exposures [27,28]. Our results for non-smokers in THIN and UK Biobank also show a cross-over pattern in lung cancer incidence by sex since 2008 and may support a role for other factors.

### 4.3 Strengths and limitations

The main advantage of a large-scale analysis of LCINS for a single nation or country, is the ability to understand any temporal changes in the context of demographic shifts and government legislation. The UK is an interesting nation for studying LCINS trends due to the dramatic shifts in employment patterns over the past 60 years together with the more recent introduction of smoke free legislation. The dataset derived from routine health records is representative of the UK suggesting our cohort is broadly representative of people reporting as non-smokers to their primary care physicians.

Like many large-scale analyses, we had to rely on self-reported smoking status to define our cohort. Further, we are relying on a GP interpretation of self-reported smoking status and the definition of a “never” smoker may differ across GPs and GP practices. Compared with data from the Health Survey for England, those who quit at a young age (< 30 years) are less likely to be recorded as an ex-smoker in THIN data relative to people who quit later in life [29]. In the UK, approximately 50 % of people are self-reported never smokers whereas the proportion for THIN using our definition was 44 %. This could reflect misclassification due to our sensitive algorithm for identifying ever smokers or that those excluded due to missing smoking data were predominantly non-smokers. The observed time trends could somewhat reflect changes in reporting accuracy but we feel this is unlikely to fully account for the sex differences.

We have analysed lung cancer cases recorded in general practice. These data have been shown to be accurate for chronic conditions but less so for acute conditions that present to hospitals [11]. In the UK, more than one third of lung cancer cases first present in the emergency hospital setting [30] and there may be some underreporting in primary care data. However, as discussed earlier, our incidence rates were similar to another large-scale UK cohort with hospital diagnosed events [24]. Although we present a large-scale analysis, LCINS is relatively rare and some estimates were imprecise and comparisons uncertain for some strata. Due to concerns over missing data and coding quality [31], we were unable to differentiate on lung cancer subtypes and confirm earlier studies on the predominance of adenocarcinoma in LCINS [32]. We cannot be certain that the lung cancer diagnosis was a primary or secondary tumour. However, a separate THIN study that reviewed full text medical records to validate small-cell lung cancer diagnoses found that only two out of 400 diagnoses were for secondary tumours [31]. The sociodemographic and urbanicity variables are derived from the UK Census for 2001 and may not be as accurate for more recent time periods. These two variables are only available from the data provider as categorical variables and the mutually adjusted estimates should be interpreted with caution. Finally, UK Biobank participants are not representative of the general population and cannot be used to provide representative disease prevalence and incidence rates. Participants tend to be less socially deprived and have much lower rates of lung cancer compared with the general population [33].

### 4.4 Conclusions

Based on our results using a large and broadly representative sample of the UK, LCINS rates between 1998 and 2018 appear relatively stable in women but have decreased quite substantially in men. Further research is needed to investigate the upward trend in LCINS incidence since 2008 for women living in the least socially deprived areas.

## Contributors

LJH, GR contributed to the study design. LJH conducted statistical analyses. LJH wrote the initial draft of the manuscript. Both authors participated in the data interpretation and contributed to the final draft of the manuscript with intellectual importance.

## Funding

Laura Horsfall is supported by a Wellcome Trust Fellowship [Grant Number 209207/Z/17/Z].

## Patient consent for publication

Not required.

## Data availability statement

The data that support the findings of this study are available from UK Biobank and The Health Improvement Network (THIN) IQVIA™ Medical Research Data. Restrictions apply to the availability of these data, which were used under license for this study.

## CRediT authorship contribution statement

**Greta Rait:** Supervision, Writing - review & editing. **Laura Horsfall:** Conceptualization, Methodology, Software, Data curation, Writing - original draft, Visualization, Investigation.

## Declaration of Competing Interest

None declared.
