# Supplementary material for: Twenty-year sociodemographic trends in lung cancer in non-smokers: A UK-based cohort study of 3.7 million people
Source: Cancer Epidemiol. 2020 Aug;67:101771. doi: 10.1016/j.canep.2020.101771 (PMC7397470; doi:10.1016/j.canep.2020.101771)
Supplement: Supplementary file 1 [file mmc1.docx]

# Appendices

The UK Biobank Resource is a prospective cohort study of over 500,000 participants aged 40–69 years and recruited between 2006–2010 from different regions of the UK[^16^](#_ENREF_16). Participants attended 22 centres with locations selected to ensure representation of people from different socioeconomic, ethnic and urban-rural backgrounds. This ongoing study collects data from questionnaires, sample assays, physical measures, genome-wide genotyping and follow-up for a wide range of health-related outcomes some of which are linked to national registers and electronic health records. UK Biobank received ethics approval from the National Health Service National Research Ethics Service (Ref 11/NW/0382).

Participants enter the cohort on the date they attended the research centre and are censored at the earliest date of lung cancer diagnosis, loss to follow-up, death or end of the follow-up period. The most recent date of complete follow-up for incident cancers at the time of analysis was 3/31/2016 for England and Wales and 10/31/2015 for Scotland. Participants with a history of lung cancer at the time of recruitment were excluded from the primary analysis of lung cancer incidence (n=512).

Prevalent and incident cancer diagnoses in UK Biobank are provided by The Health & Social Care Information Centre for participants residing in England and Wales, and the NHS Central Register for participants residing in Scotland. These national cancer registries obtain information from a range of sources including hospitals, treatment centres, hospices and nursing homes, private hospitals, general practices, death certificates, and Hospital Episode Statistics. Diagnoses are coded using the International Classification of Disease (ICD) version 9 and 10. The primary outcome is lung cancer, which we defined as malignant neoplasms of the trachea and bronchus (ICD10: C33-C34).

Table A.1: Read codes for identifying smoking status

| Read code | Status | Description | Frequency in full dataset |
| --- | --- | --- | --- |
| 1371.11 | Non/never | Non-smoker | 10874 |
| 9kn..11 | Non/never | Non-smoker annual review | 30 |
| 9kn..00 | Non/never | Non-smoker annual review - enhanced services administration | 11670 |
| 1371.00 | Non/never | Never smoked tobacco | 200994 |
| 9OO9.00 | Ever | Stop smoking monitoring delete | 44 |
| 9OO1.00 | Ever | Attends stop smoking monitor. | 65314 |
| 745H200 | Ever | Nicotine replacement therapy using nicotine inhalator | 3938 |
| 9kc0.00 | Ever | Smoking cessatn monitor template complet - enhanc serv admin | 96 |
| 9OO..11 | Ever | Stop smoking clinic admin. | 12822 |
| ZV11600 | Ever | [V]Personal history of tobacco abuse | 10 |
| 8I2I.00 | Ever | Nicotine replacement therapy contraindicated | 14 |
| Eu17400 | Ever | [X]Men & behav dis due tobacco: withdrawl state wth delirium |  |
| 8I3M.00 | Ever | Bupropion refused | 16 |
| Eu17z00 | Ever | [X]Ment & behav dis due use tobacco: unsp ment & behav dis |  |
| 8B2B000 | Ever | Issue of nicotine replacement therapy voucher | 45 |
| 8BPh.00 | Ever | Bupropion therapy | 4 |
| 9Ndf.00 | Ever | Consent given for follow-up by smoking cessation team | 1798 |
| 8B3Y.00 | Ever | Over the counter nicotine replacement therapy | 3765 |
| 8CdB.00 | Ever | Stop smoking service opportunity signposted | 5975 |
| 9OO3.00 | Ever | Stop smoking monitor default | 2066 |
| 9Ndg.00 | Ever | Declined consent for follow-up by smoking cessation team | 4997 |
| Eu17y00 | Ever | [X]Men & behav dis due to use tobacco: oth men & behav dis |  |
| 137D.00 | Ever | Admitted tobacco cons untrue ? | 1 |
| 8B3f.00 | Ever | Nicotine replacement therapy provided free | 12117 |
| 13p1.00 | Ever | Smoking status at 4 weeks | 37081 |
| 137k.00 | Ever | Refusal to give smoking status | 3039 |
| 137g.00 | Ever | Cigarette pack-years | 10985 |
| ZRBm211 | Ever | FTND - Fagerstrom test for nicotine dependence | 29 |
| 745H500 | Ever | Varenicline therapy | 240 |
| 9OOB100 | Ever | Stop smoking invitation second SMS text message | 278 |
| 13p7.00 | Ever | Smoking status at 12 weeks | 330 |
| 9hG0.00 | Ever | Excepted from smoking quality indicators: Patient unsuitable | 17833 |
| 9hG1.00 | Ever | Excepted from smoking quality indicators: Informed dissent | 59501 |
| 9kc..00 | Ever | Smoking cessation - enhanced services administration | 2609 |
| 9kc0.11 | Ever | Smoking cessation ESA monitoring template completed | 9 |
| 9OO..00 | Ever | Anti-smoking monitoring admin. | 22743 |
| 9N2k.00 | Ever | Seen by smoking cessation advisor | 277906 |
| 13p3.00 | Ever | Smoking status at 52 weeks | 1593 |
| J036400 | Ever | Tobacco deposit on teeth | 2 |
| 8I2J.00 | Ever | Bupropion contraindicated | 10 |
| 9NdY.00 | Ever | Declin cons follow-up evaluation after smoking cess interven | 306 |
| 745Hy00 | Ever | Other specified smoking cessation therapy | 2837 |
| 745H400 | Ever | Smoking cessation drug therapy | 12240 |
| 9NdV.00 | Ever | Consent given follow-up after smoking cessation intervention | 26 |
| 9OOZ.00 | Ever | Stop smoking monitor admin.NOS | 14974 |
| Eu17000 | Ever | [X]Mental & behav dis due to use tobacco: acute intoxication |  |
| Eu17100 | Ever | [X]Mental and behav dis due to use of tobacco: harmful use | 4 |
| Eu17200 | Ever | [X]Mental and behav dis due to use tobacco: dependence syndr | 4 |
| Eu17600 | Ever | [X]Mental and behav dis due to use tobacco: amnesic syndrome |  |
| Eu17500 | Ever | [X]Mental & behav dis due to use tobacco: psychotic disorder | 2 |
| 745H100 | Ever | Nicotine replacement therapy using nicotine gum | 1745 |
| H310100 | Ever | Smokers' cough | 2572 |
| 9OO8.00 | Ever | Stop smoking monitor phone inv | 12075 |
| 9OO5.00 | Ever | Stop smoking monitor 2nd lettr | 11434 |
| TJHy200 | Ever | Adverse reaction to nicotine | 20 |
| U609900 | Ever | [X]Bupropion causing adverse effects in therapeutic use | 4 |
| 9hG..00 | Ever | Exception reporting: smoking quality indicators | 1514 |
| ZRBm200 | Ever | Fagerstrom test for nicotine dependence | 2981 |
| 745H300 | Ever | Nicotine replacement therapy using nicotine lozenges | 1507 |
| 9OO4.00 | Ever | Stop smoking monitor 1st lettr | 82623 |
| 8B2B.00 | Ever | Nicotine replacement therapy | 70954 |
| 13p5.00 | Ever | Smoking cessation programme start date | 31425 |
| 8HBP.00 | Ever | Smoking cessation 12 week follow-up | 231 |
| Eu17700 | Ever | [X]Men & beh dis due tobacco: resid & late-onset psychot dis |  |
| 38DH.00 | Ever | Fagerstr test for nicotine dep | 418 |
| 745H.00 | Ever | Smoking cessation therapy | 92295 |
| ZV6D800 | Ever | [V]Tobacco abuse counselling | 697 |
| 9OO2.00 | Ever | Refuses stop smoking monitor | 11710 |
| 745H000 | Ever | Nicotine replacement therapy using nicotine patches | 15221 |
| 9OO7.00 | Ever | Stop smoking monitor verb.inv. | 4542 |
| Eu17.00 | Ever | [X]Mental and behavioural disorder due to use of tobacco | 6 |
| 8BP3.00 | Ever | Nicotine replacement therapy provided by community pharmacis | 539 |
| 13p6.00 | Ever | Carbon monoxide reading at 4 weeks | 14433 |
| 9OO6.00 | Ever | Stop smoking monitor 3rd lettr | 4067 |
| 13p2.00 | Ever | Smoking status between 4 and 52 weeks | 5966 |
| Eu17300 | Ever | [X]Mental and behav dis due to use tobacco: withdrawal state | 3 |
| 13p4.00 | Ever | Smoking free weeks | 8242 |
| 137n.00 | Ever | Total time smoked | 1021 |
| 13p5000 | Ever | Practice based smoking cessation programme start date | 4977 |
| 8I39.00 | Ever | Nicotine replacement therapy refused | 2344 |
| 9OOB200 | Ever | Stop smoking invitation third SMS text message | 29 |
| 137o.00 | Ever | Waterpipe tobacco consumption | 29 |
| 9OOB000 | Ever | Stop smoking invitation first SMS text message | 3745 |
| E023.00 | Ever | Nicotine withdrawal | 1368 |
| 13p8.00 | Ever | Lost to smoking cessation follow-up | 761 |
| 745Hz00 | Ever | Smoking cessation therapy NOS | 8005 |
| 9OO..12 | Ever | Stop smoking monitoring admin. | 44851 |
| 13p..00 | Ever | Smoking cessation milestones | 125256 |
| 9N4M.00 | Ever | DNA - Did not attend smoking cessation clinic | 32499 |
| 9OOA.00 | Ever | Stop smoking monitor.chck done | 18166 |
| 8HBM.00 | Ever | Stop smoking face to face follow-up | 2863 |
| 9NdW.00 | Ever | Consent given for smoking cessation data sharing | 783 |
| 9NdZ.00 | Ever | Declined consent for smoking cessation data sharing | 99 |
| 8B31G00 | Ever | Varenicline smoking cessation therapy offered | 46 |
| 9OOB.00 | Ever | Stop smoking invitation short message service text message | 4325 |
| 137l.00 | Ever | Ex roll-up cigarette smoker |  |
| 137K.00 | Ever | Stopped smoking | 24025 |
| 137j.00 | Ever | Ex-cigarette smoker | 393 |
| 1377.00 | Ever | Ex-trivial smoker (<1/day) | 29 |
| 137K000 | Ever | Recently stopped smoking | 18 |
| 1379.00 | Ever | Ex-moderate smoker (10-19/day) | 104 |
| 137S.00 | Ever | Ex smoker | 43309 |
| 137L.00 | Ever | Current non-smoker | 12860 |
| 137N.00 | Ever | Ex pipe smoker | 5 |
| 137A.00 | Ever | Ex-heavy smoker (20-39/day) | 120 |
| 9km..00 | Ever | Ex-smoker annual review - enhanced services administration | 67 |
| E251300 | Ever | Tobacco dependence in remission | 356 |
| 1378.00 | Ever | Ex-light smoker (1-9/day) | 68 |
| 137T.00 | Ever | Date ceased smoking | 8999 |
| 137B.00 | Ever | Ex-very heavy smoker (40+/day) | 16 |
| 137F.00 | Ever | Ex-smoker - amount unknown | 2278 |
| 9km..11 | Ever | Ex-smoker annual review | 2007 |
| 137O.00 | Ever | Ex cigar smoker | 8 |
| 67H1.00 | Ever | Lifestyle advice regarding smoking | 25082 |
| 1372.00 | Ever | Trivial smoker - < 1 cig/day | 507 |
| 1374.00 | Ever | Moderate smoker - 10-19 cigs/d | 8055 |
| 13p0.00 | Ever | Negotiated date for cessation of smoking | 71643 |
| ZRaM.00 | Ever | Motives for smoking scale | 18 |
| 6791.00 | Ever | Health ed. - smoking | 2524426 |
| 137e.00 | Ever | Smoking restarted | 1134 |
| ZRh4.00 | Ever | Reasons for smoking scale | 7 |
| 137V.00 | Ever | Smoking reduced | 252 |
| 137..11 | Ever | Smoker - amount smoked | 1674 |
| E251100 | Ever | Tobacco dependence, continuous | 48 |
| 8IAj.00 | Ever | Smoking cessation advice declined | 95395 |
| 8H7i.00 | Ever | Referral to smoking cessation advisor | 203973 |
| E251000 | Ever | Tobacco dependence, unspecified | 30 |
| 137b.00 | Ever | Ready to stop smoking | 12090 |
| 137J.00 | Ever | Cigar smoker | 378 |
| 8IEM.00 | Ever | Smoking cessation drug therapy declined | 48827 |
| 137H.00 | Ever | Pipe smoker | 422 |
| 137h.00 | Ever | Minutes from waking to first tobacco consumption | 4932 |
| 137M.00 | Ever | Rolls own cigarettes | 668 |
| 8HTK.00 | Ever | Referral to stop-smoking clinic | 99263 |
| 137G.00 | Ever | Trying to give up smoking | 72274 |
| 9ko..11 | Ever | Current smoker annual review | 3739 |
| ZRao.00 | Ever | Occasions for smoking scale | 2 |
| 137Q.11 | Ever | Smoking restarted | 505 |
| 9kf2.11 | Ever | COPD structured smoking assessment declined | 9 |
| 137P.00 | Ever | Cigarette smoker | 72100 |
| 1375.00 | Ever | Heavy smoker - 20-39 cigs/day | 6815 |
| 9kf1.11 | Ever | Referred for COPD structured smoking assessment | 4 |
| ZG23300 | Ever | Advice on smoking | 6788 |
| 1373.00 | Ever | Light smoker - 1-9 cigs/day | 5644 |
| E251z00 | Ever | Tobacco dependence NOS | 528 |
| 137c.00 | Ever | Thinking about stopping smoking | 14977 |
| E251.00 | Ever | Tobacco dependence | 9078 |
| 137d.00 | Ever | Not interested in stopping smoking | 17419 |
| E251200 | Ever | Tobacco dependence, episodic | 8 |
| SMC..00 | Ever | Toxic effect of tobacco and nicotine | 26 |
| 9NS0200 | Ever | Referral for smoking cessation service offered | 9997 |
| 8IEo.00 | Ever | Referral to smoking cessation service declined | 14052 |
| 8IEK.00 | Ever | Smoking cessation programme declined | 31430 |
| 137f.00 | Ever | Reason for restarting smoking | 212 |
| ZRh4.11 | Ever | RFS - Reasons for smoking scale | 5 |
| 8CAL.00 | Ever | Smoking cessation advice | 4663857 |
| ZV4K000 | Ever | [V]Tobacco use | 91 |
| 137P.11 | Ever | Smoker | 13916 |
| 67A3.00 | Ever | Pregnancy smoking advice | 2794 |
| 9kf1.00 | Ever | Refer COPD structured smoking assessment - enhanc serv admin | 133 |
| 1V08.00 | Ever | Smokes drugs in cigarette form | 8 |
| 8CAg.00 | Ever | Smoking cessation advice provided by community pharmacist | 1123 |
| 137Q.00 | Ever | Smoking started | 1394 |
| ZRaM.11 | Ever | MFS - Motives for smoking scale | 4 |
| 137m.00 | Ever | Failed attempt to stop smoking | 2488 |
| 1376.00 | Ever | Very heavy smoker - 40+cigs/d | 1780 |
| 137R.00 | Ever | Current smoker | 24788 |
| ZRao.11 | Ever | OFS - Occasions for smoking scale |  |
| 8IEM000 | Ever | Varenicline smoking cessation therapy declined |  |
| 8T08.00 | Ever | Referral to smoking cessation service | 4327 |
| 67H6.00 | Ever | Brief intervention for smoking cessation | 11254 |
| 9ko..00 | Ever | Current smoker annual review - enhanced services admin | 48 |
| 9kf2.00 | Ever | COPD structured smoking assessment declined - enh serv admin | 67 |
| 1372.11 | Ever | Occasional smoker | 386 |
| 8HkQ.00 | Ever | Referral to NHS stop smoking service | 32902 |
| 137C.00 | Ever | Keeps trying to stop smoking | 514 |
| 137E.00 | Ever | Tobacco consumption unknown | 103 |

Table A.2: Read codes for identifying lung cancer

| Read code | Description | Frequency in full dataset |
| --- | --- | --- |
| B22..00 | Malignant neoplasm of trachea, bronchus and lung | 12174 |
| B220.00 | Malignant neoplasm of trachea | 215 |
| B220000 | Malignant neoplasm of cartilage of trachea |  |
| B220100 | Malignant neoplasm of mucosa of trachea | 1 |
| B220z00 | Malignant neoplasm of trachea NOS | 29 |
| B221.00 | Malignant neoplasm of main bronchus | 3103 |
| B221000 | Malignant neoplasm of carina of bronchus | 154 |
| B221100 | Malignant neoplasm of hilus of lung | 488 |
| B221z00 | Malignant neoplasm of main bronchus NOS | 780 |
| B222.00 | Malignant neoplasm of upper lobe, bronchus or lung | 2477 |
| B222.11 | Pancoast's syndrome | 318 |
| B222000 | Malignant neoplasm of upper lobe bronchus | 610 |
| B222100 | Malignant neoplasm of upper lobe of lung | 3364 |
| B222z00 | Malignant neoplasm of upper lobe, bronchus or lung NOS | 305 |
| B223.00 | Malignant neoplasm of middle lobe, bronchus or lung | 335 |
| B223000 | Malignant neoplasm of middle lobe bronchus | 81 |
| B223100 | Malignant neoplasm of middle lobe of lung | 403 |
| B223z00 | Malignant neoplasm of middle lobe, bronchus or lung NOS | 47 |
| B224.00 | Malignant neoplasm of lower lobe, bronchus or lung | 1003 |
| B224000 | Malignant neoplasm of lower lobe bronchus | 265 |
| B224100 | Malignant neoplasm of lower lobe of lung | 1747 |
| B224z00 | Malignant neoplasm of lower lobe, bronchus or lung NOS | 193 |
| B225.00 | Malignant neoplasm of overlapping lesion of bronchus & lung | 90 |
| B22y.00 | Malignant neoplasm of other sites of bronchus or lung | 563 |
| B22z.00 | Malignant neoplasm of bronchus or lung NOS | 34733 |
| B22z.11 | Lung cancer | 34022 |
| B811.00 | Carcinoma in situ of trachea | 24 |
| B812.00 | Carcinoma in situ of bronchus and lung | 2877 |
| B812000 | Carcinoma in situ of carina of bronchus | 49 |
| B812100 | Carcinoma in situ of main bronchus | 129 |
| B812200 | Carcinoma in situ of upper lobe bronchus and lung | 94 |
| B812300 | Carcinoma in situ of middle lobe bronchus and lung | 12 |
| B812400 | Carcinoma in situ of lower lobe bronchus and lung | 47 |
| B812z00 | Carcinoma in situ of bronchus or lung NOS | 475 |
| Byu2000 | [X]Malignant neoplasm of bronchus or lung, unspecified | 159 |

Figure A.1: Predicted age-adjusted incidence of lung cancer in non-smokers at three time points in the past 20-years by sex at birth and UK country.

|  | Age-adjusted IRR (95%CI) | P-value | Predicted age adjusted incidence per 10,000 PY | IRR per year (95%CI) | IRR per year (95%CI) | P-value |
| --- | --- | --- | --- | --- | --- | --- |
| Women | | | | | | |
| English regions |  |  |  | 1998-2007 | 2008-2018 |  |
| East Midlands | 1.03 (0.73 to 1.47) |  | 1.51 (1.02 to 1.99) | 1.00 (0.90 to 1.10) | 1.03 (0.86 to 1.23) |  |
| East of England | 0.91 (0.72 to 1.16) |  | 1.33 (1.07 to 1.59) | 1.07 (0.96 to 1.18) | 1.02 (0.93 to 1.12) |  |
| London | 1.00 (ref) |  | 1.46 (1.26 to 1.66) | 0.99 (0.93 to 1.04) | 1.04 (0.98 to 1.10) |  |
| North East | 1.21 (0.88 to 1.66) |  | 1.76 (1.25 to 2.26) | 0.85 (0.78 to 0.93) | 1.11 (0.99 to 1.24) |  |
| North West | 1.34 (1.10 to 1.63) |  | 1.95 (1.67 to 2.23) | 0.97 (0.92 to 1.01) | 1.01 (0.97 to 1.06) |  |
| South Central | 1.07 (0.88 to 1.30) |  | 1.56 (1.34 to 1.78) | 1.02 (0.95 to 1.09) | 1.05 (0.99 to 1.12) |  |
| South East Coast | 0.88 (0.72 to 1.08) |  | 1.29 (1.09 to 1.49) | 1.02 (0.93 to 1.13) | 1.01 (0.95 to 1.07) |  |
| South West | 0.97 (0.78 to 1.21) |  | 1.41 (1.17 to 1.66) | 1.01 (0.94 to 1.08) | 0.93 (0.85 to 1.02) |  |
| West Midlands | 0.93 (0.76 to 1.15) |  | 1.36 (1.14 to 1.58) | 1.00 (0.92 to 1.08) | 1.03 (0.94 to 1.12) |  |
| Yorkshire & Humber | 1.27 (0.96 to 1.67) | 0.0036 | 1.85 (1.41 to 2.29) | 0.91 (0.83 to 0.99) | 1.08 (0.94 to 1.24) | 0.055 |
| Men | | | | | | |
| English regions |  |  |  | 1998-2007 | 2008-2018 |  |
| East Midlands | 1.52 (1.12 to 2.06) |  | 2.61 (1.99 to 3.24) | 0.89 (0.76 to 1.04) | 1.13 (0.90 to 1.41) |  |
| East of England | 1.09 (0.85 to 1.40) |  | 1.87 (1.56 to 2.19) | 0.98 (0.91 to 1.06) | 0.89 (0.80 to 1.00) |  |
| London | 1.00 (ref) |  | 1.72 (1.4 0to 2.04) | 0.89 (0.82 to 0.97) | 1.00 (0.93 to 1.08) |  |
| North East | 1.68 (1.14 to 2.47) |  | 2.88 (1.89 to 3.87) | 0.85 (0.77 to 0.94) | 1.10 (0.99 to 1.22) |  |
| North West | 1.48 (1.13 to 1.95) |  | 2.55 (2.04 to 3.06) | 0.91 (0.86 to 0.96) | 1.00 (0.92 to 1.08) |  |
| South Central | 0.97 (0.75 to 1.26) |  | 1.67 (1.36 to 1.98) | 0.92 (0.87 to 0.99) | 0.96 (0.87 to 1.06) |  |
| South East Coast | 0.88 (0.68 to 1.14) |  | 1.51 (1.24 to 1.79) | 0.90 (0.85 to 0.95) | 0.94 (0.88 to 1.00) |  |
| South West | 1.10 (0.82 to 1.49) |  | 1.9 0(1.45 to 2.34) | 0.87 (0.82 to 0.93) | 1.01 (0.92 to 1.11) |  |
| West Midlands | 1.09 (0.81 to 1.45) |  | 1.87 (1.44 to 2.29) | 0.87 (0.83 to 0.91) | 0.98 (0.90 to 1.07) |  |
| Yorkshire & Humber | 1.58 (1.11 to 2.24) | 0.0001 | 2.72 (1.91 to 3.52) | 1.03 (0.92 to 1.16) | 1.04 (0.92 to 1.19) | 0.0217 |

Table A.3: Age-adjusted incidence rates and incidence predictions for lung cancer in non-smokers by UK geographic region and sex at birth

IRR=Incidence rate ratio

*Wald test for categorical variable

**Wald test for multiplicative interaction term between variable and calendar year

#
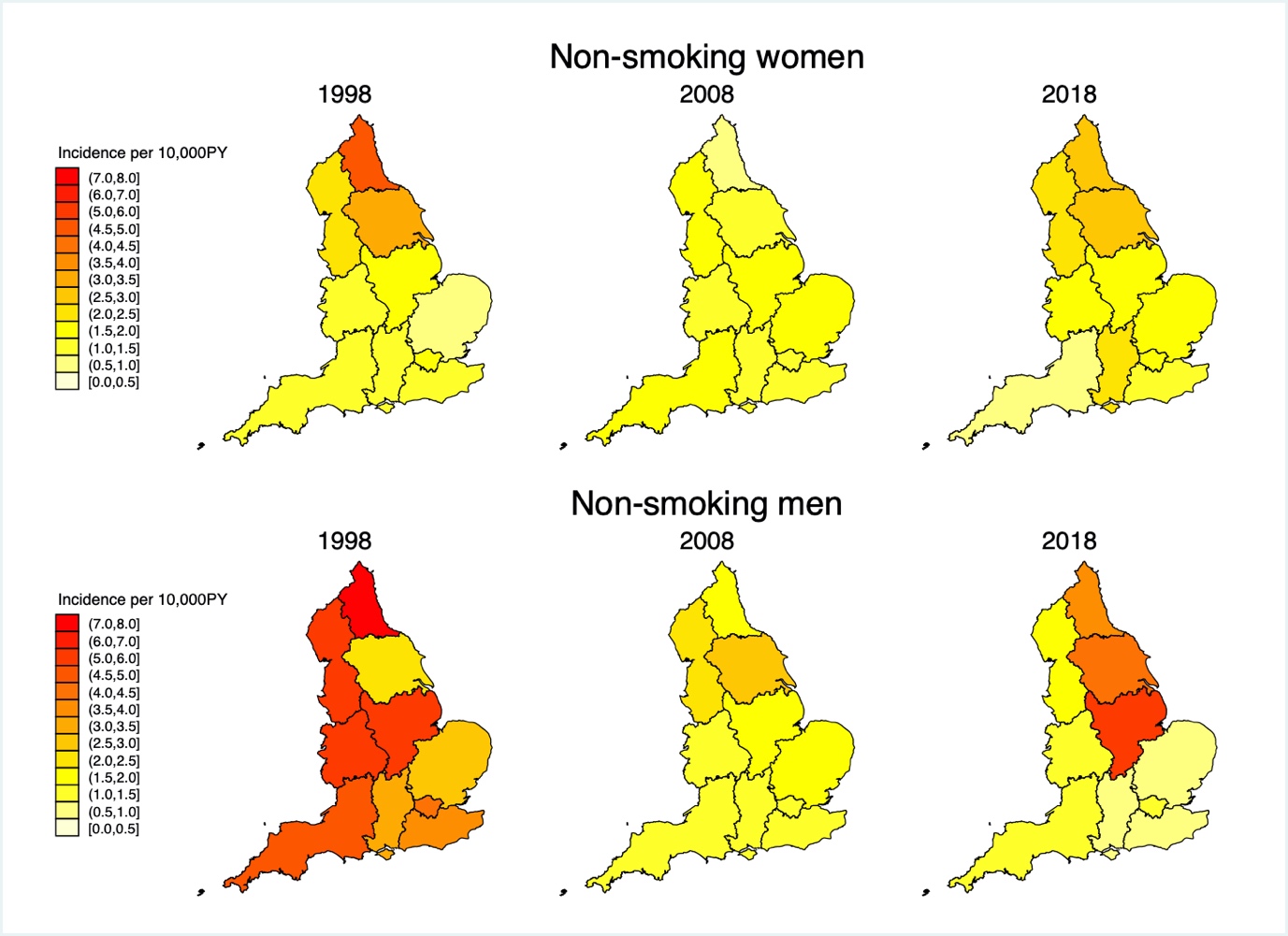


Figure A.2: Predicted age-adjusted incidence of lung cancer in non-smokers at three time points in the past 20-years by sex at birth and region of England.


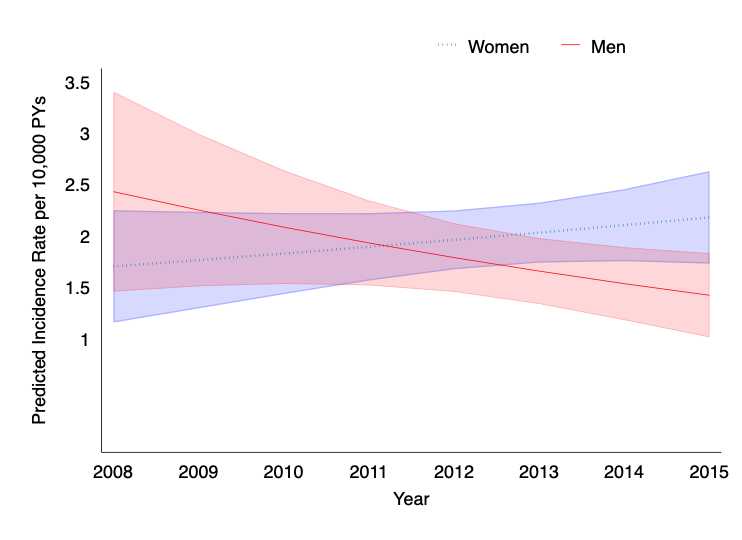


Figure A.3: Predicted age-adjusted incidence of lung cancer in never smokers in UK Biobank by calendar year.
